# Improving Tracking of Selective Attention in Hearing Aid Users: The Role of Noise Reduction and Nonlinearity Compensation

**DOI:** 10.1523/ENEURO.0275-24.2025

**Published:** 2025-02-14

**Authors:** Johanna Wilroth, Emina Alickovic, Martin A. Skoglund, Carine Signoret, Jerker Rönnberg, Martin Enqvist

**Affiliations:** ^1^ Automatic Control, Department of Electrical Engineering, Linköping University, Linköping 581 83, Sweden; ^2^ Eriksholm Research Centre, Snekkersten DK-3070, Denmark; ^3^ Disability Research Division, Linnaeus Centre HEAD, Department of Behavioural Sciences and Learning, Linköping University, Linköping 581 83, Sweden

**Keywords:** EEG, hearing aids, noise, noise reduction algorithms, nonlinearity compensation, temporal response functions

## Abstract

Hearing impairment (HI) disrupts social interaction by hindering the ability to follow conversations in noisy environments. While hearing aids (HAs) with noise reduction (NR) partially address this, the “cocktail-party problem” persists, where individuals struggle to attend to specific voices amidst background noise. This study investigated how NR and an advanced signal processing method for compensating for nonlinearities in Electroencephalography (EEG) signals can improve neural speech processing in HI listeners. Participants wore HAs with NR, either activated or deactivated, while focusing on target speech amidst competing masker speech and background noise. Analysis focused on temporal response functions to assess neural tracking of relevant target and masker speech. Results revealed enhanced neural responses (N1 and P2) to target speech, particularly in frontal and central scalp regions, when NR was activated. Additionally, a novel method compensated for nonlinearities in EEG data, leading to improved signal-to-noise ratio (SNR) and potentially revealing more precise neural tracking of relevant speech. This effect was most prominent in the left-frontal scalp region. Importantly, NR activation significantly improved the effectiveness of this method, leading to stronger responses and reduced variance in EEG data and potentially revealing more precise neural tracking of relevant speech. This study provides valuable insights into the neural mechanisms underlying NR benefits and introduces a promising EEG analysis approach sensitive to NR effects, paving the way for potential improvements in HAs.

## Graphical abstract

**Figure:**
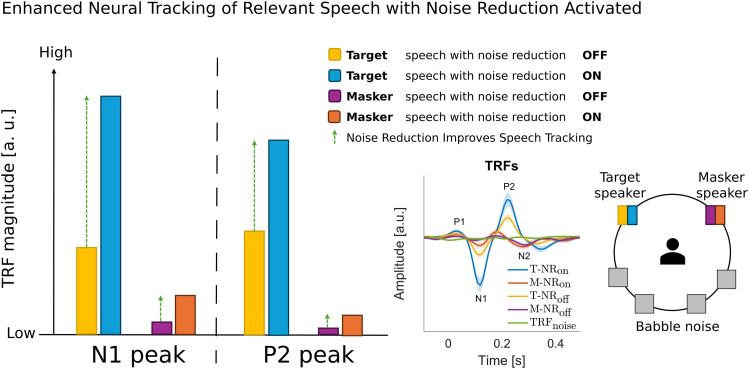
Visual Abstract

## Significance Statement

Understanding how HAs with noise reduction (NR) improve selective auditory attention in noisy environments is crucial for future advancements. This study investigated the neural effects of NR in hearing-impaired listeners using Electroencephalography (EEG). We observed significantly enhanced neural responses (N1 and P2 peaks) to target speech with NR activated, suggesting improved speech tracking in frontal and central scalp regions. The advanced signal processing method also compensated for nonlinearities in EEG data, improving the signal-to-noise ratio (SNR) and revealing more precise neural tracking, particularly in the left-frontal scalp region. This study sheds light on the neural mechanisms behind NR benefits and introduces a promising EEG analysis method sensitive to NR effects, paving the way for optimizing future HAs.

## Introduction

Electroencephalography (EEG)-based hearing research has grown rapidly in recent years. This focus is well-founded, considering hearing loss ranks as the third most prevalent chronic health condition among the elderly population (Meneses-Barriviera et al., 2013). Studies have shown that hearing loss greatly affects social interactions and even contributes to depression (Boi et al., 2012; Cosh et al., 2019; Lawrence et al., 2019). While HAs demonstrably improve the quality of life for hearing-impaired people (Cohen et al., 2004; Chisolm et al., 2007; Tsakiropoulou et al., 2007; Lotfi et al., 2009; Lacerda et al., 2012), challenges remain, especially in noisy listening environments. Modern HAs partially address this challenge by incorporating noise reduction (NR) algorithms that aim to attenuate unwanted background noise (Fiedler et al., 2021).

Another major challenge for HAs is the “cocktail party problem” (Cherry, 1953): the ability to selectively amplify the attended stimuli while suppressing unattended stimuli. Evidence suggests this ability deteriorates with hearing loss (Bronkhorst, 2000; Marrone et al., 2008; Mcdermott, 2009). Consequently, HA users often experience both a louder environment due to sound amplification, and increased difficulty distinguishing between attended and unattended speakers compared to those with normal hearing.

This study investigates two crucial factors affecting neural tracking of relevant speech: the influence of NR algorithms on enhancing attention to relevant speech and reducing interference from irrelevant environmental noise, and the impact of physiological noise in EEG data. We assessed these factors in participants with hearing impairment who were instructed to attend to one of two simultaneously presented speech streams (target) while ignoring the other (masker), and additional environmental noise. Environmental “noise” refers to unwanted background sounds that NR algorithms in HAs aim to diminish. However, the term “noise” also applies to the inherent limitations of EEG data. Here, physiological noise refers to electrical activity unrelated to auditory processes that contaminate the signal recorded by the EEG electrodes on the scalp. This type of noise results in a low SNR and requires specific processing techniques for effective analysis.

Neural tracking can be assessed using temporal response functions (TRFs), which are linear filters estimated at the sensor level in response to a stimulus such as continuous speech (Alickovic et al., 2019; Di Liberto et al., 2020; Brodbeck et al., 2023). Unlike event-related potentials (ERPs), TRFs are not time-locked to specific events. Yet, their peaks align with the similar stages of the speech processing observed in ERPs, revealing how different aspects of the stimulus drive neural activity over time (Crosse et al., 2016). These responses to attended stimulus typically exhibit three main components: P1 around 50 − 70 ms (related to early detection of auditory stimuli), N1 around 80 − 120 ms and P2 around 150 − 275 ms (higher-order auditory processing, modulated by attention) (Key et al., 2005; Lightfoot, 2016; Brodbeck and Simon, 2020; Smith and Ferguson, 2024).

Prior research has used a backward TRF model to evaluate the effect of different NR algorithms on neural tracking of relevant speech (Alickovic et al., 2020, 2021; Andersen et al., 2021). Results found that active NR enhanced the neural representation of relevant speech while suppressing the representation of background noise. However, these models are limited by their anti-causal nature, making it difficult to interpret their results in terms of temporal and spatial dynamics. To overcome this limitation, the first contribution of this study is to use a forward TRF model to predict EEG activity from the speech stimuli. This approach has not been previously investigated using data collected under different NR settings. The effectiveness of the NR algorithms is evaluated using established metrics, which constitutes the first contribution of this study: amplitude and latency of the N1 and P2 peaks (reflecting speech processing) and TRF variance (consistency of EEG responses).

The second contribution of this study involves investigating noisy EEG data using a binning-based nonlinearity detection and compensation method presented in Wilroth et al. (2024). Applied to EEG data from 30 participants (Andersen et al., 2021), our results replicated compensation patterns observed previously in one-subject analysis (Wilroth et al., 2024), particularly in the left-frontal scalp region. Finally, an SNR analysis of the EEG data showed a significant improvement after the nonlinearity compensation.

## Materials and Methods

### Experimental dataset

EEG data were recorded from 32 subjects (*M* = 19) with mild-to-moderate hearing loss using a 64-channel BioSemi ActiveTwo system at a sampling rate of 1024 Hz. Due to incomplete data, two subjects were excluded from our analysis. The experiment took place in a soundproof room. The subjects sat facing two loudspeakers positioned in front of them (at ±30°), playing different Danish news clips of neutral content. In each trial, the subject was instructed to focus on one loudspeaker and ignore the other. Four additional loudspeakers were located behind the subject, playing background noise consisting of 16-talker babble at an SNR of +3 dB. Each trial started with 5 s of background noise, followed by 33 s of news clips and concluded with a two-choice question regarding the content of the attended speech. Subjects performed 40 trials for each hearing aid condition, with NR either activated (NR_on_) or deactivated (NR_off_), leading to a total of 80 trials per subject. This dataset has been used for various analyses, and a complete description of the experimental setup can be found in Andersen et al. (2021). The study was reviewed and approved by the Swedish Ethics Review Authority (DNR: 2022-05129-01).

The two speech streams (target, masker) and the two hearing aid noise reduction settings (on, off) result in four different analysis conditions. These will henceforth be denoted “T-NR_on_ ,” “M-NR_on_ ,” “T-NR_off_ ,” and “M-NR_off_ .”

### Data preprocessing

To preprocess the speech streams, we first extracted the envelopes using the absolute value of the Hilbert transform. We applied a 6th order Butterworth filter for band-pass filtering between 1 − 8 Hz, followed by downsampling to 100 Hz. To mitigate edge effects, we removed the first and last seconds of the data. A delay of 49.1 ms was observed between the actual stimuli presented over the audio signal path (RME stimulus soundcard → ProTools → Dante system → Speaker 2.2m from microphone → DPA microphone → RME recording soundcard) and event markers fed directly into the EEG recordings (RME stimulus soundcard → RME recording soundcard). This delay was accounted for in the analysis.

For EEG data preprocessing, we re-referenced the data to the two mastoid channels. Next, we filtered out the 50 Hz line noise and applied the 6th-order Butterworth filter for bandpass filtering at 0.5 − 70 Hz. Channels with artifacts (on average 19 per subject) were identified through manual inspection and compensated for by interpolating neighboring channels. Independent component analysis was performed to identify and remove artifacts such as eye blinks, heart beats and muscle movements (Oostenveld et al., 2011). On average, 17.2 components per subject were removed. Final preprocessing steps included a bandpass filter of 1 − 8 Hz using a 6th order Butterworth filter, downsampling to 100 Hz and matching data to the speech envelopes. More detailed information can be found in Andersen et al. (2021) and Alickovic et al. (2020, 2021).

### Workflow overview

**Figure 1.**
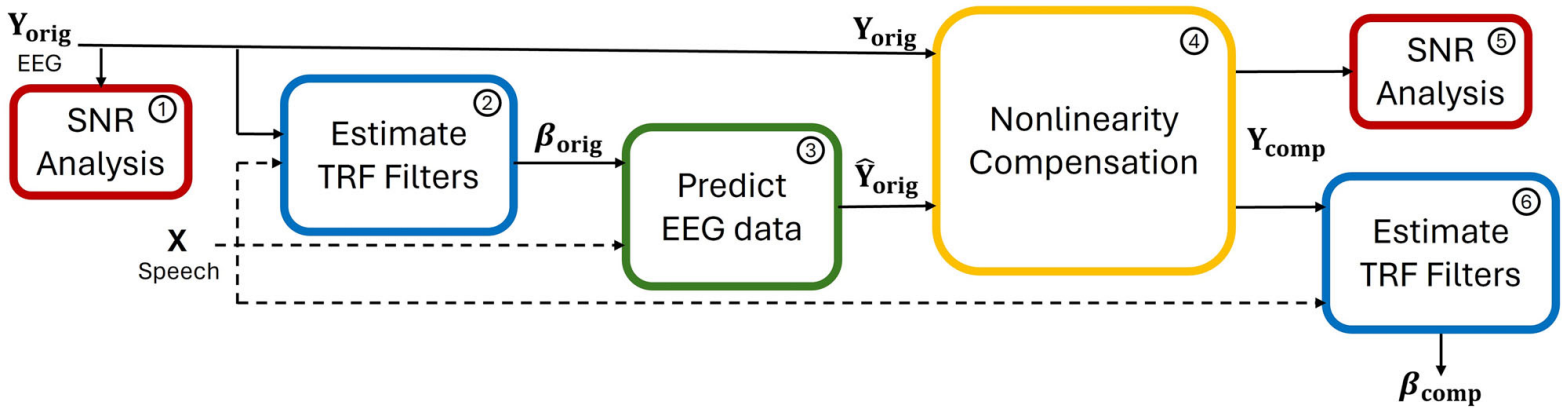
Workflow overview. The steps are as follows: (1) Calculate the SNR of the original (measured) EEG data **Y**_orig_. (2) Estimate the TRFs **β**_orig_ from original EEG data **Y**_orig_ and the speech envelopes **X**^T^ (target) and **X**^M^ (masker). (3) Compute the predicted original EEG data ${\hat{\text{Y}}}_{\mathrm{orig}}$ from the TRFs **β**_orig_ and the speech envelopes **X**^T^ and **X**^M^. (4) Compute the compensated EEG data **Y**_comp_ from **Y**_orig_ and ${\hat{\text{Y}}}_{\mathrm{orig}}$ with the nonlinearity detection and compensation method. (5) Compute the SNR of the compensated EEG data **Y**_comp_. (6) Estimate the TRFs **β**_comp_ from compensated EEG data **Y**_comp_ and the speech envelopes **X**^T^ and **X**^M^.

The workflow used in this article, presented in Figure 1, is divided into six steps:

1. Compute the SNR of the measured EEG data **Y**_orig_.
2. Estimate the TRFs **β**_orig_ from original EEG data **Y**_orig_ and the speech envelopes **X**^T^ (target) and **X**^M^ (masker).
3. Compute the predicted original EEG data ${\hat{\text{Y}}}_{\mathrm{orig}}$ from the TRFs **β**_orig_ and the speech envelopes **X**^T^ and **X**^M^.
4. Compute the compensated EEG data **Y**_comp_ from **Y**_orig_ and ${\hat{\text{Y}}}_{\mathrm{orig}}$ with the nonlinearity detection and compensation method.
5. Compute the SNR of the compensated EEG data **Y**_comp_.
6. Compute the TRFs **β**_comp_ from compensated EEG data **Y**_comp_ and the speech envelopes **X**^T^ and **X**^M^.

All notation is collected in Table 1.

**Table 1.** Table of Notations

Notation	Description
**Y**_orig_	Measured EEG data
${\hat{\text{Y}}}_{\mathrm{orig}}$	Predicted EEG data
**Y**_comp_	EEG data after compensation
${\hat{\text{Y}}}_{\mathrm{comp}}$	Predicted EEG data after compensation
**X** ^T^	Target speech envelope
**X** ^M^	Masker speech envelope
***β***	Estimated TRFs
**E**	Noise matrix
NR_on_	Noise reduction activated
NR_off_	Noise reduction deactivated
T-NR_on_	Target speech with noise reduction *on*
M-NR_on_	Masker speech with noise reduction *on*
T-NR_off_	Target speech with noise reduction *off*
M-NR_off_	Masker speech with noise reduction *off*

### Temporal response functions (TRFs)

A forward linear model, also known as an encoding model or a TRF model (Alickovic et al., 2019), is used to predict the EEG response to a specific speech stimulus. The model estimates the relationship between a known stimulus input, *x*(*t*) (e.g., speech envelope), and measured output *y*(*t*, *n*) (e.g., EEG data), at each time point *t* (from 1 to *T*) and for each channel *n* (from 1 to *N*). The TRF model is given by

$$y(t,n)=\sum_{\tau }\beta (\tau ,n)x(t-\tau )+\epsilon (t,n),$$

where *β*(*τ*, *n*) is the channel-specific TRF across a range of time lags *τ*, and ϵ(t,n) is the channel-specific residual response not accounted for by the model. The *K* number of time lags, *τ* = [ − 100, 400] ms and sampling frequency *fs*, are chosen to include the N1 and P2 components.

A common approach to estimate the linear filters is to use a coordinate descent technique referred to as the boosting algorithm (David et al., 2007; Brodbeck et al., 2023). This sparse estimation method starts by initializing the TRF at each channel with zeros. During each iteration, small fixed values are incrementally added until the mean square error (MSE) no longer decreases. The incremental additions to the TRF are derived from a dictionary of basis elements, such as Hamming windows (Kulasingham and Simon, 2023). We use the boosting algorithm implemented in the Python toolkit Eelbrain (Brodbeck et al., 2023) for the estimation of TRFs. Subsequently, the predicted EEG $\hat{y}(t,n)$ for the channel *n* is given as follows:

$$\hat{y}(t,n)=\sum_{\tau }\beta (\tau ,n)x(t-\tau ),$$

and can be estimated using the convolve function available in the Eelbrain toolkit (Brodbeck et al., 2023).

The TRF model 1 can analogously be parameterized and solved for multiple channels simultaneously using matrix notation

Y=βX+E,

where $Y\in R^{N\times T}$, $\beta \in R^{N\times K}$, $X\in R^{K\times T}$ and $E\in R^{N\times T}$.

### Binning-based nonlinear compensation

**Figure 2.**
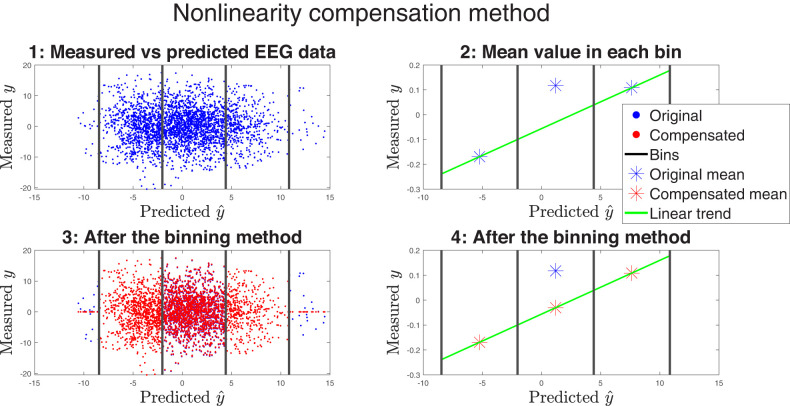
Illustration of our binning-based nonlinearity detection and compensation method applied to channel *C*_*z*_. **Top-left:** Each blue dot represent the EEG sample at time *t* in the coordinate system $\left(\text{predicted EEG, measured EEG})=(\hat{y}(t,n),y(t,n)\right)$. The 20 smallest and largest predicted samples are considered outliers and excluded from the further analysis. The remaining samples are then divided into three equally spaced bins. **Top-right:** The average value in each bin is computed and a line is fitted between the two values in the outer bins. **Bottom-left:** All samples in the middle bin (red) are shifted such that the new middle-bin average falls on the fitted line. **Bottom-right:** This adjustment results in a more accurate representation of the relationship between the predicted and measured EEG data.

Figure 2 illustrates our binning-based nonlinear compensation method. We first compute TRFs using the boosting algorithm with actual EEG data **Y**_orig_ and speech envelopes **X**. Predicted EEG data ${\hat{Y}}_{\mathrm{orig}}$ is then computed using Equation 2, which takes the TRF model and speech envelopes as input. The top left subplot of Figure 2 shows all EEG samples at time *t* for subject *s*, trial *i* and channel *n*. The coordinate system is given by $\left(\hat{y}(t,n),y(t,n)\right)$. Here, the 20 smallest and largest predicted samples are considered outliers and excluded from the further analysis. The remaining samples are then divided into three equally spaced bins. Next, the average of the measured EEG in each bin is calculated. A line is then fitted between the average values in the two outer bins, as shown in the top right subplot of Figure 2. The nonlinear compensation is applied to all samples within the middle bin (bottom left) such that the new average of the middle bin falls on the fitted line (bottom right). This process is repeated for all channels, trials and subjects, resulting in a compensated EEG dataset denoted **Y**_comp_. A more detailed explanation of the algorithm can be found in Wilroth et al. (2024).

#### Nonlinearity analysis

The difference between the original and the compensated mean values in the middle bin (Fig. 2, bottom right) represents the residual resulting from the assumed linear trend. Figure 2 illustrates a scenario where the original mean is higher than the compensated mean. This is categorized as a *positive*, or concave, nonlinearity. Conversely, a scenario where the original mean is lower than the compensated mean signifies a *negative*, or convex, nonlinearity. These residuals serve as a measure of the nonlinearities detected in the EEG data. In this study, we focus on the magnitude of the nonlinearities, leaving the exploration of specific concave and convex patterns of the nonlinearities for future work.

We obtained a channel-specific measure of the nonlinearity by averaging across all subjects and trials for each of the four experimental conditions. Visualization through topoplots revealed interesting differences between the four conditions. We conducted paired *t*-tests to determine statistically significant differences in nonlinearity magnitude between corresponding channels across the four conditions. Here, the paired observations are represented by the difference in nonlinearity values between the corresponding channels. To account for multiple comparisons and reduce the risk of false positives, we applied the Bonferroni correction, see e.g., Haynes (2013). This involved multiplying each *p*-value by the total number of channels *N*. Channels with corrected *p*-values lower than the significance level (*α* = 0.05) are considered to have statistically significant differences in the magnitude of nonlinearity. The significance level of *α* = 0.05 is used in all our statistical tests, unless otherwise stated.

### Evaluation methods

**Figure 3.**
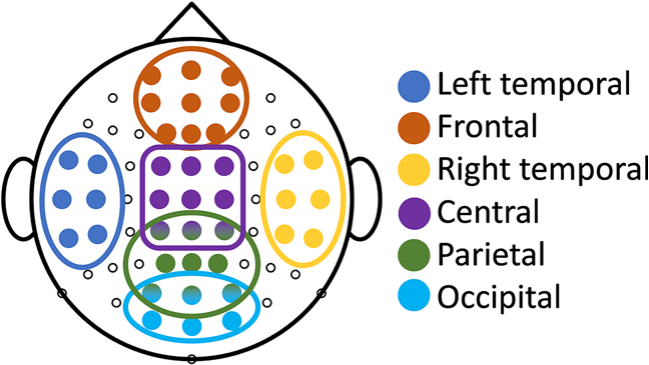
Illustration of the EEG channels included in each of the six channel groups denoted: left temporal, frontal, right temporal, central, parietal and occipital.

While the grand average TRF computed for each condition by averaging the TRFs across all subjects, trials, and channels, is a common approach, it can mask important spatial information by neglecting the contribution of individual channels. To address this, we also analyzed TRFs for six anatomically distinct channel clusters based on their location on the scalp (illustrated in Fig. 3): “left temporal,” “frontal,” “right temporal,” “central,” “parietal” and “occipital.” Most analyses in this study involve averaging the results across channels within each group.

Two evaluation methods are employed to assess the performance of our model and the impact of NR: SNR analysis (see SNR analysis section) and TRF analysis, which includes the N1 and P2 components, variance, and statistical significance compared to a noise TRF (see TRF analysis section).

#### SNR analysis

EEG data is inherently noisy, and improving the SNR could potentially lead to more accurate models. Therefore, we analyzed the difference in SNR between compensated and original EEG data. A positive difference indicates an improvement in SNR after applying the compensation method. To calculate the SNR difference, we first computed noise level in the EEG data. A noise vector of the same size as the EEG data was initialized, squared element-wise, and averaged over the number of samples, denoted *κ* in 4. Similarly, the signal power for both the original and compensated EEG data was calculated. We achieved this by taking the absolute values of the original (**Y**_orig_) and compensated (**Y**_comp_) EEG signals, squaring them element-wise, and averaging. Finally, the difference SNR_diff_ was calculated as follows:

$${\text{SNR}}_{\mathrm{diff}}=10\text{log}\frac{{\text{Y}}_{\mathrm{comp}}}{\kappa }-10\text{log}\frac{{\text{Y}}_{\mathrm{orig}}}{\kappa }.$$

#### TRF analysis

We analyzed the TRFs averaged across 30 subjects and all trials for each of the four conditions (T-NR_on_, M-NR_on_, T-NR_off_ and M-NR_off_). First, we compared the grand average TRFs. Then, we evaluated the averaged TRFs within each channel group, as defined in Figure 3. The evaluation focused on three aspects of TRFs:

1. **Amplitude and latency of the N1 and P2 peaks**: A larger amplitude indicates a stronger neural response, while a shorter latency suggests a faster response. Typically, the N1 peak occurs between 80 − 120 ms and the P2 peak occurs between 150 and 275 ms after stimulus onset (Key et al., 2005; Smith and Ferguson, 2024).
2. **Variance**: Ideally, the variance of the TRFs should be low, indicating a consistent response across the trials. We compared the variance of the original and the compensated TRFs for each channel group to assess the impact of compensation on response consistency.
3. **Comparison to noise level**: To ensure the observed effects were not due to noise, we compared each of the four conditions with a noise TRF. The $\mathrm{TR}F_{\mathrm{noise}}$ were computed by mismatching the target speech trial with the EEG speech trial and the evaluation method was a permutation test as described in the following section.

#### Permutation test based on cluster statistics

Permutation tests, implemented in the FieldTrip toolbox (Oostenveld et al., 2011; Petersen et al., 2016; FieldTrip, 2024), were conducted between the TRFnoise condition and each of the four conditions (T-NR_on_, M-NR_on_, T-NR_off_, and M-NR_off_). This analysis was performed on both the grand average TRFs and the TRFs from each of the six channel groups. It was performed accordingly:

1. For each time lag and channel, dependent-samples *t*-tests were carried out between $\mathrm{TR}F_{\mathrm{noise}}$ and each of the four conditions. This resulted in a matrix of *t*-values.
2. Next, adjacent time samples and a set number of neighboring channels with *p*-values, based on the obtained *t*-values, less than 0.05 were grouped together to form clusters. In the case of grand average TRFs, all channels were used in the dependent-samples *t*-tests, and at least three neighboring channels were required to form a cluster. For the channel-group analysis, the channels within each group were used, and at least one neighboring channel was needed to form a cluster.
3. The sum of the single-sample *t*-values within each cluster were computed and compared to the sum of *t*-values from permuted clusters. The permuted clusters were generated by randomly assigning time electrode samples to one of the two compared conditions in 1,000 iterations (Maris and Oostenveld, 2007).
4. To be considered a significant cluster, the sum of its *t*-values needs to be greater than the 95% percentile of the permutation distribution. This corresponds to a one-sided *p*-value < 0.05.

#### Behavioral performances

The responses to the two-choice questions presented to subjects after each trial reflect how well they followed the target speech. For these two-choice questions (*c* = 2), the theoretical chance level is 50%. The percentage of correct answers was computed as both as a trial-averaged individual accuracy and a grand average for both NR conditions. The empirical chance level (statistically significant threshold) for a sample size of *n* = 30 subjects, at *α* = 0.05, was calculated in MATLAB St(α)=binoinv(1−α,n,1/c)×100/n=66.33%, as used in previous studies (Combrisson and Jerbi, 2015; Alickovic et al., 2021). To assess the effect of NR on behavioral performance, a two-sample paired *t*-test was performed between the grand averages of NR_off_ and NR_on_ conditions.

### Code accessibility and data availability

There are ethical restrictions on sharing the data set. The consent given by participants at the outset of this study did not explicitly detail sharing of the data in any format; this limitation is keeping with EU General Data Protection Regulation, and is imposed by the Research Ethics Committees of the Capital Region in the country it was collected. Due to this regulation, and the way data were collected with a low number of participants where it is not possible to fully anonymize the dataset, it is not possible for us to share the dataset. Data requests can be sent to a non-author contact point (Contact for data requests: Claus Nielsen, clni@eriksholm.com.).

10.1523/ENEURO.0275-24.2025.d1 Code Download Code, ZIP file.

**Figure 4.**
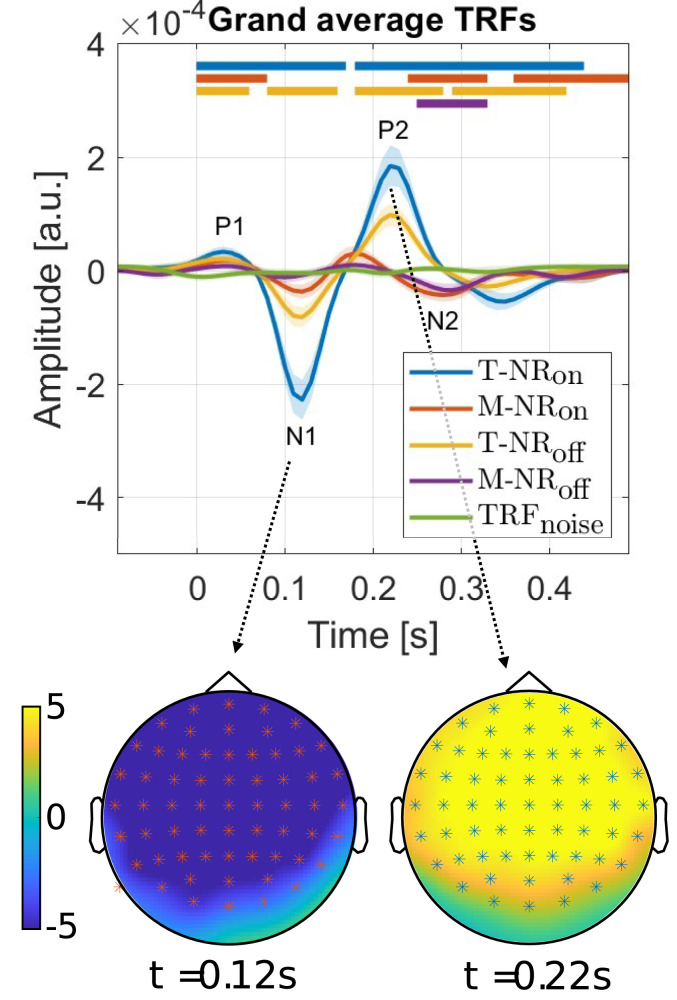
**Top**: Grand average TRFs across 30 subjects and 64 EEG channels for target and masker envelopes with NR algorithms either switched *on* or *off*. The variance of the TRFs is presented as a lighter shade of the corresponding color. Horizontal lines indicate time intervals where the TRF from each condition significantly differs from $\mathrm{TR}F_{\mathrm{noise}}$, obtained from the cluster-based permutation test from the latency *t* = 0 s(*p* < 0.05). **Bottom**: Topoplots from T-NR_on_ for the N1 peak at *t* = 0.127 s and P2 peak at *t* = 0.22 s. EEG channels marked with asterisk (*) show statistically significant differences compared to $\mathrm{TR}F_{\mathrm{noise}}$.

**Figure 5.**
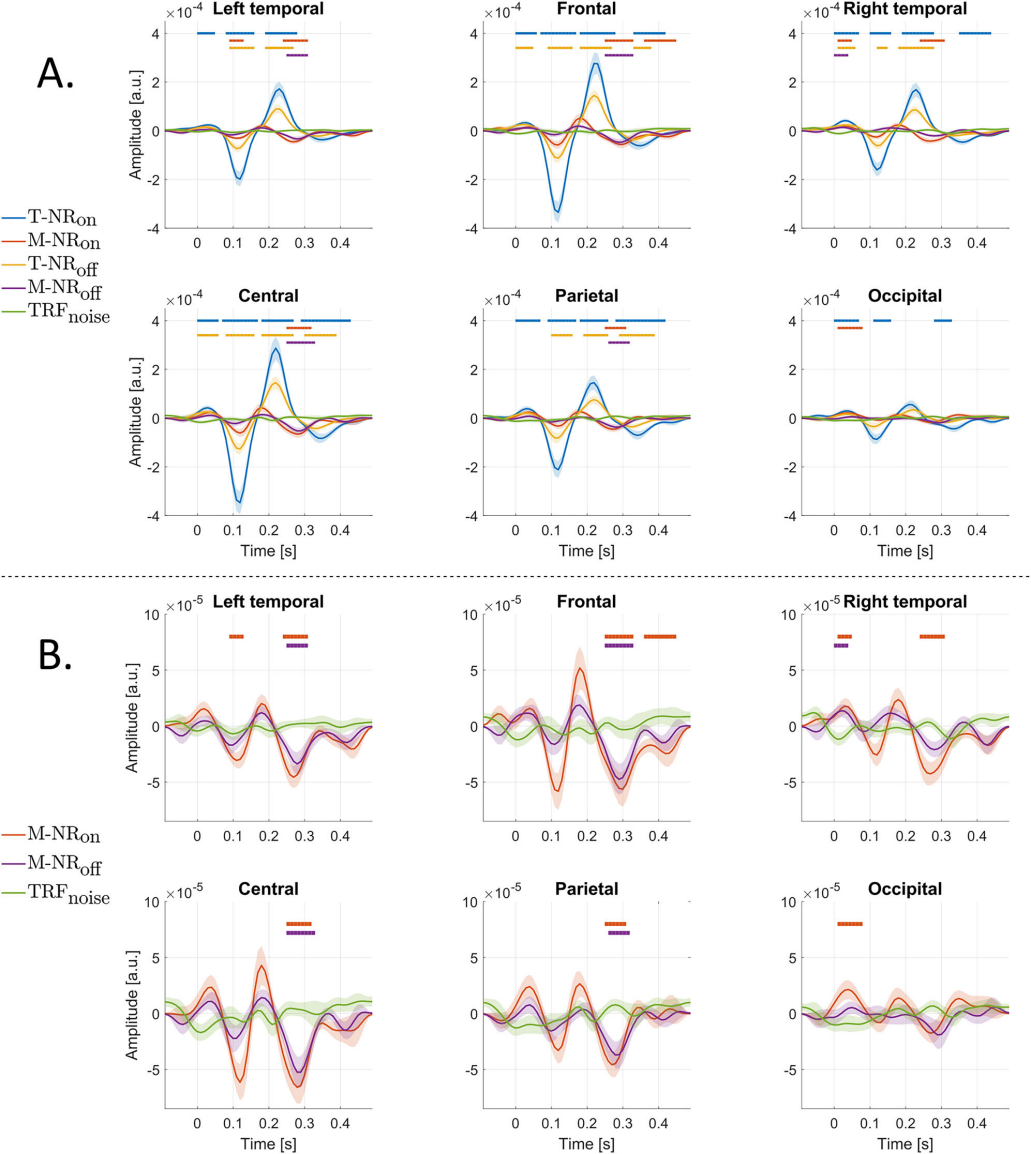
Grand average TRFs across 30 participants and the channel groups (see Fig. 3). The noise reduction (NR) algorithms were either switched *on* or *off*. Horizontal lines indicate a significant difference between the TRF from each condition and a $\mathrm{TR}F_{\mathrm{noise}}$, obtained from the cluster-based permutation test from the latency *t* = 0 s (*p* < *α*). ***A***, Grand average TRFs for the four conditions T-NR_on_, M-NR_on_, T-NR_off_, and M-NR_off_. The N1 and P2 peaks are visible for the target speech in all channel groups, with the largest amplitudes observed in the frontal and central EEG channels. **B**, Grand average TRFs for the masker speech envelope for NR_on_ and NR_off_, along with the $\mathrm{TR}F_{\mathrm{noise}}$. M-NR_on_ shows more significant parts of the TRF compared to M-NR_off_, indicating that NR affects the processing of masker speech.

**Figure 6.**
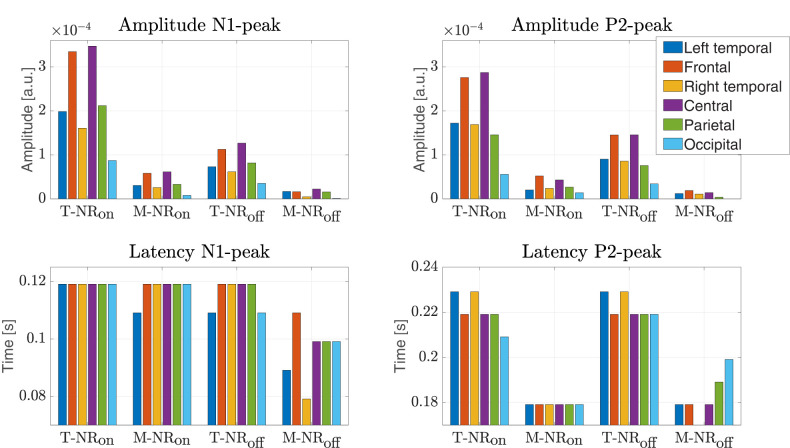
Analysis of the N1 (most negative deflection within *t* = [0.08, 0.12] s) and P2 (most positive deflection within *t* = [0.18, 0.25] s) peaks obtained from the TRFs presented in Figure 5*A*. The four conditions (T-NR_on_, M-NR_on_, T-NR_off_, M-NR_off_) across six scalp regions are evaluated. **Top:** The amplitude of the peaks. **Bottom:** The latency of the peaks.

**Figure 7.**
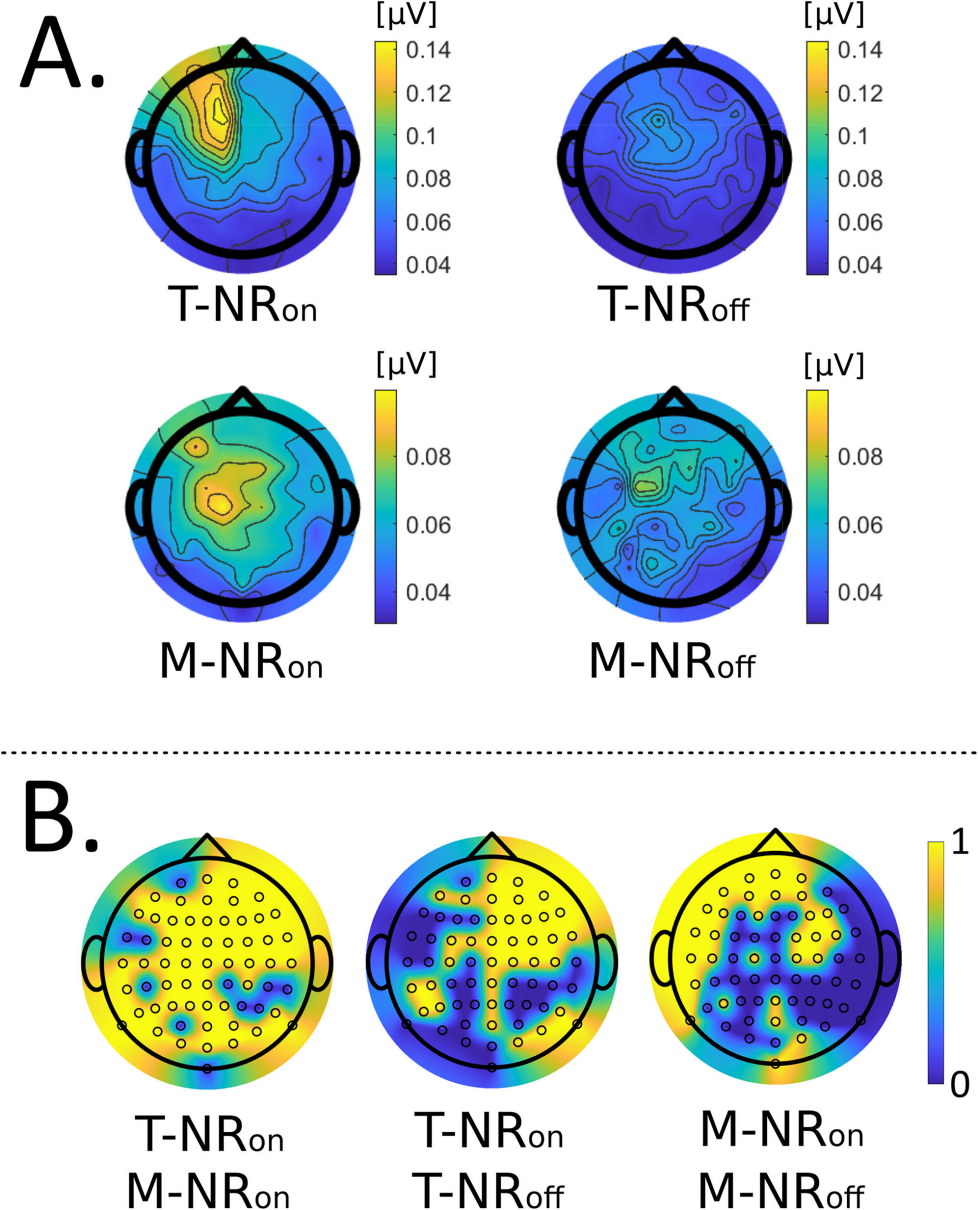
Measure of nonlinearity obtained from the middle bin residuals in the nonlinearity detection and compensation method. ***B***, Average residuals (compensation) [*μ*V] over 30 participants for target and masker envelopes with NR_on_ (top) and NR_off_ (bottom). The largest compensation for each condition was obtained with the target speech, and NR_on_ acquired a larger compensation compared to NR_off_. (A) *t*-test for statistically significance (*p* < *α*), between the combinations:T-NR_on_-M-NR_on_ (difference between target and masker), T-NR_on_-T-NR_off_ and M-NR_on_-M-NR_off_ (difference between NR_on_ and NR_off_). Yellow channels (value 1): statistically significant and blue channels (value 0): not statistically significant.

**Figure 8.**
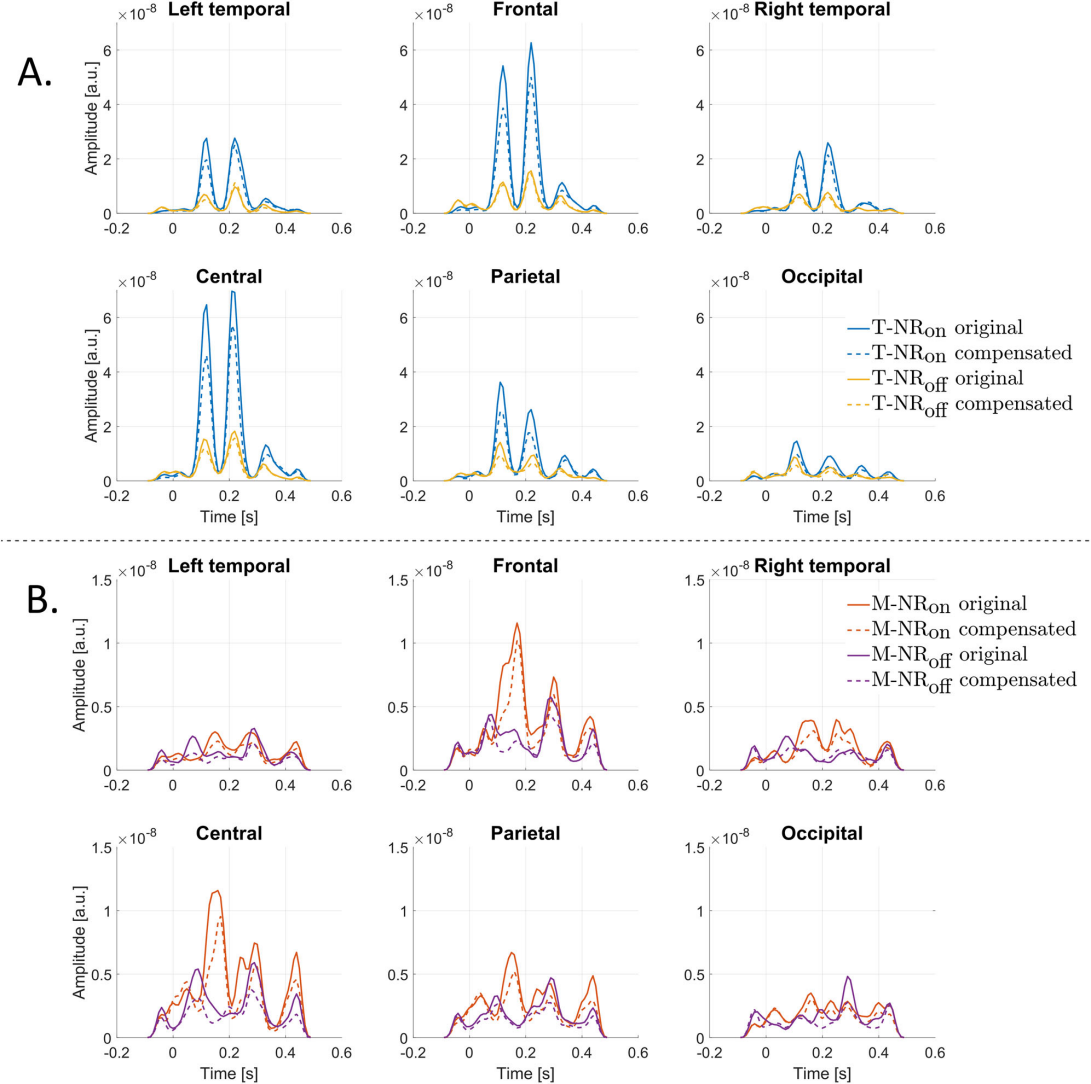
TRF variance for target and masker speech envelopes. Solid line represents original EEG data, and the dashed line represents compensated EEG data. Variance amplitudes differ between target and masker speech streams, as indicated on the *y*-axis. ***A***, TRF variance for the target envelope. The largest variances align with the N1 and P2 peaks around 120 ms and 220 ms, respectively, where a large peak amplitude (Fig. 6) results in a large peak variance. The compensated EEG data show reduced TRFs variance (dashed lines) for both NR_on_ and NR_off_ conditions across all channel groups. ***B***, TRF variance for the masker envelope. The variance amplitude is lower compared to the target envelope, consistent with the TRF amplitudes in Figure 6. The largest variances are observed in the frontal and central channel groups at the P2 peak.

**Figure 9.**
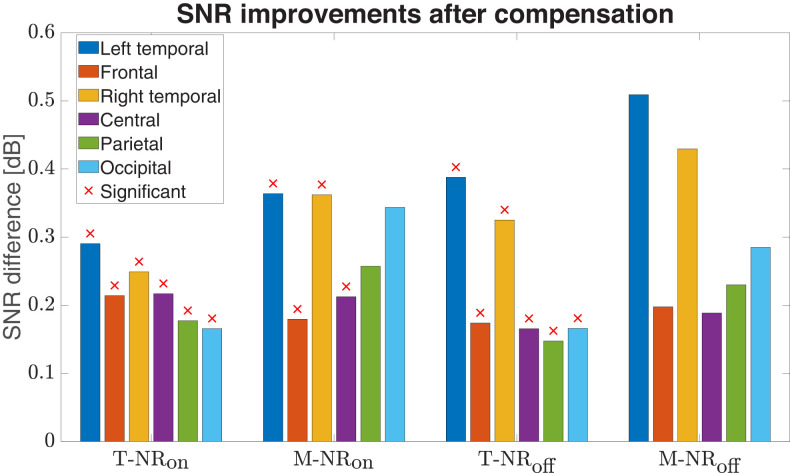
SNR differences between the compensated and the original EEG data for the four conditions (T-NR_on_, M-NR_on_, T-NR_off_, M-NR_off_) across six channel groups (Fig. 3). A positive difference indicates an improvement in SNR after applying the nonlinearity compensation method. A Bonferroni corrected *t*-test revealed that most SNR differences (marked with a red cross) for T-NR_on_, M-NR_on_, and T-NR_off_ were statistically significant (*p* < *α*).

**Figure 10.**
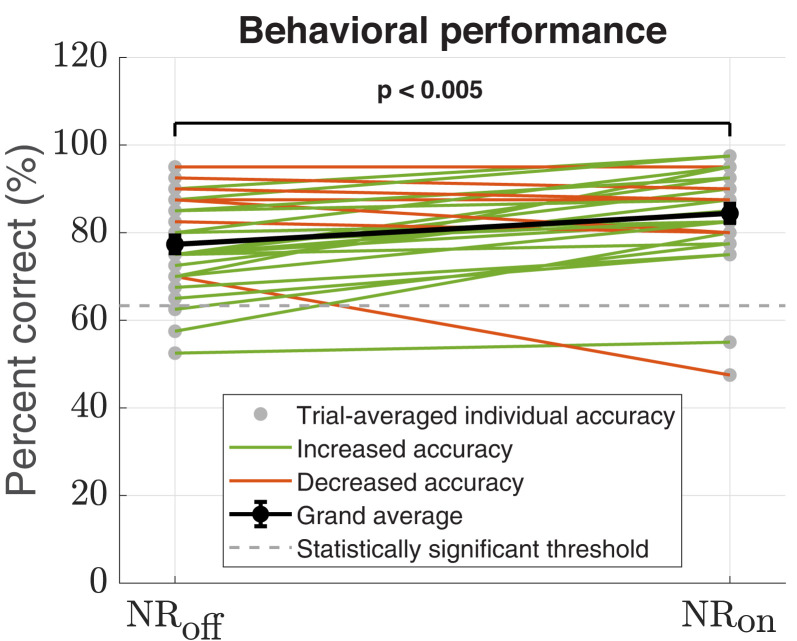
Behavioral performance for the conditions NR_off_ and NR_on_. The black dot and error bar for each condition represents the grand average ± standard deviation over all subjects and all trials. A paired-sample *t*-test revealed a significant difference (*p* = 0.003) between the two conditions, indicating that NR activated improves behavioral performance. Each gray dot represents the trial-average individual percentage of correct answers in the two-choice question. The diagonal lines connect the two points from the same subject under the two NR conditions, and the colors represent an increased (green) or a decreased (red) accuracy when NR was activated. The gray-dashed line represents the threshold for statistical significance ( 63.33%).

The TRF data and code to replicate Figures 4–10 in this study, and the script to run the binning-based nonlinearity compensation method is available in a GitHub repository (GitHub repository: https://github.com/JohannaWil/NonlinearCompensationEEG) and as Extended Data.

## Results

### TRF analysis

The grand average TRFs for all participants (*n* = 30), trials and channels, computed using original EEG data **Y**_orig_ are presented in Figure 4. The data is separated into four conditions based on target and masker speech, with NR either on or off, denoted as T-NR_on_, M-NR_on_, T-NR_off_, and M-NR_off_. Horizontal lines mark significant differences (*p* < *α*) between the TRFs of each condition and a noise-based TRF (denoted $\mathrm{TR}F_{\mathrm{noise}}$), obtained through the cluster-based permutation test from the latency *t* = 0 s. The significant time intervals include [0–0.17, 0.18–0.44] s (T-NR_on_), [0–0.08, 0.24–0.33, 0.36–0.49] s (M-NR_on_), [0–0.06, 0.08–0.16, 0.18–0.28, 0.29–0.42] s (T-NR_off_) and [0.25–0.33] s (M-NR_off_). The bottom part of the figure shows topoplots of the N1 peak at *t* = 0.12 s and P2 peak at *t* = 0.22 s for the T-NR_on_ condition. The channels marked with an asterisk (*) are statistically different from the corresponding $\mathrm{TR}F_{\mathrm{noise}}$ channels (*p* < *α*), showing widespread activity across the scalp.

To investigate how TRFs vary across scalp regions, we analyzed TRFs averaged over 6 channel groups: frontal, left temporal, right temporal, central, parietal, and occipital (presented in Fig. 3). Figure 5*A* shows the average TRFs for each group. Horizontal lines indicate significant difference between the TRF from each condition and $\mathrm{TR}F_{\mathrm{noise}}$, obtained from the cluster-based permutation test from the latency *t* = {0 s(*p* < *α*). Significant differences were observed for the target speech in the time intervals of [0–0.06, 0.09–0.16, 0.18–0.27, 0.33–0.38] s for the frontal and central groups, and in the intervals of [0.10–0.16, 0.19–0.26, 0.29–0.39] s for the parietal group. Notably, for T-NR_on_ and T-NR_off_, the intervals of [0.12–0.14] s and [0.19–0.25] s showed overlap across all groups except occipital, highlighting consistent patterns in TRF variations in response to the conditions.

Figure 5*B* compares the masker speech TRFs for the two NR conditions against $\mathrm{TR}F_{\mathrm{noise}}$, generated by mismatching the trials for the EEG data with the trials for the target speech. The results show that the peaks for both NR conditions are larger compared to the noise level across all channel groups with the NR_on_ condition yielding larger responses than NR_off_. In particular, the frontal and central channel groups show distinct N1 and P2 peaks above the noise level. Cluster-based permutation tests revealed more significant differences for M-NR_on_ compared to M-NR_off_.

Following the TRF analysis (Fig. 6), we further analyzed the N1 (most negative deflection within *t* = [0.08, 0.12] s) and P2 (most positive deflection within *t* = [0.18, 0.25] s) peaks for each condition across different scalp regions (channel groups). The top panels show peak amplitudes, with the largest target speech responses observed in the frontal and central regions, consistent with Figure 4. Both N1 and P2 amplitudes were reduced in the NR_off_ conditions, suggesting that the NR algorithms influences these responses.

The bottom panels of Figure 6 show N1 and P2 peak latencies. For N1 (bottom left), the latency is notably shorter in the left temporal channel region for M-NR_on_ and T-NR_off_ conditions compared to T-NR_on_, with a *t*-test comparing the left temporal latency of M-NR_on_ and T-NR_off_ with the left temporal latency of T-NR_on_ resulting in a *p*-value of 0.0489. For P2 (bottom right), the latency is longer for target speech in the left and right temporal regions compared to other scalp regions (*p*-value 0.0182). No significant latency differences were found between the two NR conditions for masker speech.

### Nonlinearity compensation

The difference between the original (blue) and the compensated (red) mean values in the middle bin (Fig. 2, bottom right) represents the residual resulting from the assumed linear trend. The magnitude of this residual (compensation) can be viewed as a measure of the nonlinearities detected in the EEG data. The average nonlinearity compensations [*μ*V] for 30 participants in four conditions—target and masker speech with NR either on or off, denoted as T-NR_on_, M-NR_on_, T-NR_off_ and M-NR_off_—are presented in Figure 7*A*. The most significant compensation is observed in the left-frontal scalp region for the condition T-NR_on_ (top left). In contrast, the M-NR_on_ condition (top right) exhibits a smaller compensation without a clear dominance in the left-frontal area. The two bottom plots show the condition with NR_off_, where the magnitude of the averaged nonlinearity compensation is smaller compared to T-NR_on_ (note that the scale between NR_on_ and NR_off_ are different). However, the pattern for the target speech is similar for both NR_on_ and NR_off_, where the largest compensation is located in the left region.

Three specific condition combinations deserve closer analysis: T-NR_on_-M-NR_on_ (target vs. masker), T-NR_on_-T-NR_off_ and M-NR_on_-M-NR_off_ (comparing NR_on_ and NR_off_). Channel-specific *t*-tests were conducted for these combinations, with the Bonferroni correction applied to control for multiple comparisons and reduce the risk of false positives. The results are shown in Figure 7*B*, where yellow channels indicate statistically significant differences between the two evaluated conditions (*p* < *α*). A majority of channels showed statistically significant differences when comparing T-NR_on_ condition with both M-NR_on_ (left, 53/64) and T-NR_off_ (middle, 34/64). However, fewer channels were statistically different when comparing M-NR_on_ and M-NR_off_ (right, 29/64), primarily in the left scalp region.

#### Variance analysis

The variance from the TRFs for T-NR_on_/T-NR_off_ (top) and M-NR_on_/M-NR_off_ (bottom) speech envelopes is displayed in Figure 8. The solid lines indicate the variance from the original EEG data, while the dashed lines indicate the variance for the compensated EEG data. The highest variances were obtained around the N1 and P2 peaks across all channel groups, with the frontal and central channel groups showing higher variance compared to the other groups. This indicates that larger peak amplitudes, as shown in Figure 6, correspond to larger variances. A similar trend is shown when comparing target and masker speeches, with the latter having smaller amplitude variances.

#### SNR analysis

An SNR analysis of the four conditions (T-NR_on_, T-NR_off_, M-NR_on_, M-NR_off_) and six channel groups is presented in Figure 9. Each bar shows the difference between the SNR from the compensated EEG data (after nonlinearity compensation) and the SNR from the measured EEG data (before nonlinearity compensation). All conditions for all channel groups show a positive SNR difference, indicating an improvement in SNR after applying the nonlinearity compensation method. The red cross signifies that the improvement is statistically significant (*p* < *α*) with a Bonferroni corrected *t*-test. Although large, especially for the left and right temporal channel groups, the SNR differences for M-NR_off_ were not statistically significant. Hence there is a disparity when switching on the NR, since most channel groups for M-NR_on_ resulted in a statistically significant SNR differences. Left and right temporal channel groups also produced the largest SNR differences for the target speech with both NR scenarios. Here, the larger improvements in these two channel groups for NR_off_ indicate that the nonlinearity compensation method might have a larger impact on the SNR compared to NR_on_.

### Impact of NR on behavioral performance

The percentage of correct answers on the two-choice questions, as shown in Figure 10, demonstrate that the participants were able to focus on the target speech in both NR_on_ and NR_off_ conditions. The gray-dashed line marks the statistically significant threshold, calculated to be St(α)=63.33%. The grand average ± standard deviation (%) for each NR condition was 77.33 ± 1.89 (NR_off_) and 84.42 ± 2.05 (NR_on_). A paired-sample *t*-test revealed a significant difference between the NR_off_ and NR_on_ conditions, *t*(29) = −4.06, *p* = 0.0003 (two-tailed, *α* = 0.05), indicating that NR activated improved the behavioral performance.

## Discussion

Our results highlight two key findings. First, when NR is activated, the TRF analysis in Figure 5*A* showed stronger neural responses in the frontal and central scalp regions, suggesting improved encoding of target speech. This aligns with previous research on the effects of hearing aid signal processing on neural speech tracking, suggesting that NR improves the neural representation of target speech while reducing the representation of background noise during selective attention tasks (Alickovic et al., 2020, 2021; Andersen et al., 2021). Interestingly, these same scalp regions also exhibited enhanced responses to masker speech when NR was activated, consistent with Alickovic et al. (2020, 2021) and Andersen et al. (2021). This aligns with perceptual load theory (Lavie and Tsal, 1994; Lavie, 1995), which proposes that when attentional resources are plentiful (low load), some processing of irrelevant stimuli (distractors) such as masker speech can occur. This reasoning may also be supported by the cognitive spare capacity, which argue that since the processing of target will use less cognitive resources in case of NR_on_, there is some cognitive resources left to process the masker (Rudner and Lunner, 2014). In this context, by reducing cognitive load, NR enables the brain to allocate more resources to process even unattended speech, thereby explaining the observed activity for masker speech. In other words, the stronger the masker representation the better, as long as the target representation is the strongest (compared to the masker). For higher cognitive load, on the other hand, a study found a depressed brain stem activity in a visual, letter-based, n-back odd-ball listening task (Sörqvist et al., 2012). This kind of modulation was also revealed in a later study with the same experimental setup (Sörqvist et al., 2016), but now examining cortical activity. The authors found that especially for high cortical load, primary auditory cortical area activity was reduced. Thus, for cross-modal competition between target and masker, masker activity is reduced when load is high. This would not necessarily imply that within-modality competition would follow the same pattern. Individual working memory capacity would presumably determine at which parametric level of distraction that working memory capacity per se enhances both target and masker processing, with high loads shielding the processing of the focal task and spending relatively less processing on the masker.

Second, the nonlinear compensation method introduced in this study indicates that NR and attention have impact on the amount of nonlinear components in the EEG data. Our results yielded the largest compensation for T-NR_on_ in the left-frontal region, indicating that nonlinearities were most prominent for electrodes in this specific scalp area. Notably, the NR and subsequent nonlinear compensation led to an overall improvement in the SNR of the EEG data.

### TRF analysis

The grand average TRFs (Fig. 4) reveal three characteristic neural components (P1, N1, and P2), typically observed in response to auditory stimulus, with latencies corresponding to previous studies. In both target conditions (T-NR_on_ and T-NR_off_), the peak latencies presented, as expected, larger amplitudes in the T-NR_on_ condition, suggesting enhanced neural tracking of target speech when NR is activated (Alickovic et al., 2020, 2021; Andersen et al., 2021; Fiedler et al., 2021).

Our findings show that the target speech consistently elicited stronger responses than masker speech, aligning with previous research that demonstrates cortical responses to a mixture of speakers are predominantly driven by target speech (Mesgarani and Chang, 2012; Golumbic et al., 2013; O’sullivan et al., 2015; Fuglsang et al., 2017; Brodbeck et al., 2020; Kurthen et al., 2021; Commuri et al., 2023; Orf et al., 2023; Carta et al., 2024). Across all groups (Fig. 5*A*), particularly with NR activated, responses to target speech were most pronounced in the frontal and central EEG channels. This aligns with previous studies reporting dominant frontal scalp activity for envelope TRFs (Crosse et al., 2016), centro-frontal enhancements (Fiedler et al., 2019; Carta et al., 2023, 2024), and enhancement in central region during speech-in-noise tasks (Muncke et al., 2022).

Interestingly, masker speech exhibited a prominent positive component (P2 peak) in frontal EEG channels at the latency of around 180 ms for M-NR_on_, accompanied by a second negative deflection (N2 peak) around 280 ms, observed across all scalp regions except occipital (Fig. 5*B*). The N2 peak was observed in both NR conditions. This is consistent with prior EEG studies in competing-talker environments, which found that masker and neutral speech elicited smaller, earlier P2-peaks (around 180 ms) and N2-peaks (around 250 ms), while target speech elicited a stronger P2 peak around 200 ms (Orf et al., 2023). This antipolar relationship, where the masker speech shows an N2 deflection at latencies similar to the target P2 peak, was also observed in Fiedler et al. (2019). In their study, the EEG responses showed P1-N1-P2 components for target speech in fronto-central region, while masker speech, especially at difficult SNRs, led to an additional N2 component. While both the target and masker speech streams were presented at the same SPL in our study, the presence of 16-talker babble noise at an SNR of +3 dB created a challenging listening environment that impeded the perception of target speech, likely contributing to the observed N2 component (Fig. 5*B*). This is consistent with previous studies, such as Fiedler et al. (2019), which have shown that under challenging listening conditions, the brain actively suppresses these irrelevant sounds. However, the scalp distribution of this N2 component differs, as Fiedler et al. (2019) promoted fronto-parietal region, while our findings are statistically significant also in central, left, and right temporal regions.

Furthermore, a recent study (Carta et al., 2024) investigated neural encoding of phonological information (that is, phonological categories and phonetic boundaries) in HI listeners during a competing-talker scenario using an experimental setup similar to ours, demonstrating stronger neural responses to target speech compared to masker speech, consistent with our findings. However, this study also showed significant neural encoding of phoneme onsets for both target and masker speech, suggesting that HI individuals may be more susceptible to distractions by irrelevant sounds due to increased processing of masker speech details. Suggesting that their difficulty in focusing on a specific speaker may arise from an overly robust neural encoding of phonological details for both attended and ignored sounds. Given that normal-hearing individuals typically do not process masker speech envelope at a linguistic level (Aydelott et al., 2015; Brodbeck et al., 2018), future studies should investigate how different NR algorithms and hearing aid settings influence the higher-order processing of masker speech (e.g., envelopes, phonemes, words). These findings can inform the development of objective measures of speech comprehension that can be used in clinical settings to assess hearing and in HAs to measure speech understanding.

In summary, the N1 and P2 peak amplitudes and latencies (Fig. 6) indicate that the amplitudes for both peaks were larger for the target speech compared to the masker speech, consistent with previous studies (Mesgarani and Chang, 2012; Golumbic et al., 2013; O’sullivan et al., 2015; Fuglsang et al., 2017; Brodbeck et al., 2020; Kurthen et al., 2021; Commuri et al., 2023; Orf et al., 2023), and an amplitude difference was observed between the two NR conditions, with larger responses for NR_on_ than NR_off_, indicating a positive effect of the noise reduction algorithm.

### Nonlinearity compensation

The average nonlinearity compensations were assessed (Fig. 7*A*), revealing the largest compensation in the left-frontal scalp region, particularly in channels AF3, F1, and F3 during the T-NR_on_ condition. This finding is noteworthy, as these channels are positioned over the left side of the prefrontal cortex (PFC), which is critical for executive functions such as working memory, attention control, and decision-making (Fuster, 2015). In contrast, the M-NR_on_ condition exhibited a smaller compensation without a clear dominance in the left frontal area. Similarly, the NR_off_, condition exhibited smaller compensations compared to T-NR_on_, though T-NR_off_ also showed the largest compensation in the left region.

Statistical analysis (Fig. 7*B*) revealed significant channel differences in nonlinearity compensations between the target and masker speech in most channels, including the left frontal channels that could be associated with working memory functions (Meyer et al., 2015; Gehrig et al., 2019; Signoret et al., 2020). Comparing the two NR conditions for the target speech revealed a similar pattern of nonlinearity compensation, though with a more left-central distribution and smaller amplitudes in T-NR_off_ condition. Notably, the channels F1 and F3, near the left PCF, did not show significant differences between the two NR conditions. For the masker speech, both conditions (M-NR_on_ and M-NR_off_) showed the greatest compensation in the left-frontal scalp region, with a statistical difference between the two conditions for channels AF3 and F1.

Interestingly, the largest compensation–indicating the greatest magnitude of nonlinearites in the EEG data–was observed for the T-NR_on_ condition, where the sound clarity was most favorable for the attention task. This finding aligns with the Ease of Language Understanding (ELU) model (Rönnberg et al., 2013, 2019), which posits that listeners allocate additional cognitive resources to process less clear signals. With the clearer target speech in the NR_on_ condition (Fig. 4), attending the target speech required less effort. In contrast, the (NR_off_ and masker speech) involved more signal degradation, making it harder for the incoming signal to match the incoming signals with the phonological representations in semantic long-term memory. This mismatch necessitates a postdictive mechanism in working memory that compensates for the degradation and reconstructs the degraded signal into a coherent representation for the listener. This process is slow and effortful, likely leading to increased fatigue (Pichora-Fuller et al., 2016; Rönnberg et al., 2019, 2022). This is reflected in the TRFs (Fig. 4) for the T-NR_off_ condition, which has a lower amplitude compared to T-NR_on_. Our results (Fig. 7*A*) also show that T-NR_off_ has a smaller and more central compensation compared to T-NR_on_. Likewise, the masker speech for both NR conditions has smaller TRF amplitudes and nonlinear compensations.

The ELU model is further supported by behavioral data (Fig. 10) indicating how well the participants understood the target speech. Participants reported a better performance when NR was enabled (NR_on_) compared to when it was disabled (NR_off_), indicating that NR facilitated the processing of the target speech. When NR was disabled (NR_off_), participants likely faced greater challenges attending to the intended auditory information, which could have increased listening effort and fatigue. This interpretation aligns with the ELU model, indicating that NR_on_ improves behavioral performance. Furthermore, existing research on effectiveness of NR schemes in HAs supports these findings (Lunner et al., 2020), with studies suggesting that NR can positively influence neural representation of speech stimuli (Alickovic et al., 2020, 2021; Andersen et al., 2021), improve memory for target speech (Ng et al., 2015), and reduce listening effort and fatigue (Wendt et al., 2017; Fiedler et al., 2021; Shahsavari Baboukani et al., 2022). Notably, the observed improvement in the behavioral performances with NR_on_ across most trials suggests the potential for using neural data to predict behavioral responses on a trial-by-trial basis. Although this was not investigated in the present study, it could be an interesting direction for future research.

### Variance and SNR analysis

The analysis of EEG signals, characterized by weak amplitudes and inherent nonlinearity (Puthankattil et al., 2010; Crosse et al., 2016; Mesik and Wojtczak, 2023), revealed significant insights into the effectiveness of nonlinearity compensation methods. Our findings indicated that applying this method improved the signal-to-noise ratio (SNR) across all conditions, as evidenced by lower variance in the compensated data (Fig. 8) and positive SNR differences (Fig. 9), particularly in the left and right temporal regions, which initially exhibited the lowest SNR. This improvement helps support previous research that targeted optimizing or enhancing SNR in EEG data (James et al., 1997; Hauk et al., 2002; Moran and Soriano, 2018). The M-NR_off_ condition did not show any SNR enhancement, suggesting that the original EEG signals were too disrupted to benefit from compensation. This highlights the critical role of maintaining signal clarity for effective auditory processing and underscores the challenges inherent in analyzing EEG data under less-than-ideal conditions.

### Concluding remarks

This study investigated two dimensions of noise: “environmental” noise related to background sounds and “physiological” noise referring to electrical activity not related to auditory processes that contaminates the signal recorded by EEG sensors on the scalp. We compared how different levels of environmental noise, manipulated through different NR settings in HAs (NR_off_ vs NR_on_), influenced a selective auditory attention task. TRF analysis showed larger N1 and P2 peaks when NR was activated, indicating that the NR algorithm effectively reduced environmental noise, with the most prominent effects observed in the frontal and central channel groups.

The physiological noise aspect was addressed using the nonlinearity detection and compensation method. This approach addressed some of the nonlinear components in the measured EEG data, enhancing the SNR and reducing variance. The improvements observed across most channel groups in the T-NR_on_, M-NR_on_ and T-NR_off_ conditions indicate that compensating for physiological noise can lead to clearer and more reliable EEG signals. The most evident compensation, which indicates increased nonlinearity activity, was concentrated in the left frontal scalp region, reflecting increased nonlinearity activity. Furthermore, the observed variability in compensation between the two NR settings (NR_off_ vs NR_on_) underscores the importance of activating NR for optimizing auditory attention tasks in challenging listening environments. Overall, these findings help us understand how different types of noise affect auditory processing and point to ways to improve hearing aid technologies for real-world listening situations.

## Synthesis

Reviewing Editor: Nicholas J. Priebe, The University of Texas at Austin Decisions are customarily a result of the Reviewing Editor and the peer reviewers coming together and discussing their recommendations until a consensus is reached. When revisions are invited, a fact-based synthesis statement explaining their decision and outlining what is needed to prepare a revision will be listed below. The following reviewer(s) agreed to reveal their identity: NONE.

SYNTHESIS

Both reviewers agree that the authors have improved the manuscript substantially, including making positive changes to the manuscript structure, improvements to the figure legends, and the inclusion of additional behavioral data. Three minor issues were raised in review.

Issue 1

The behavioral data raises the possibility that the neural data could be used to predict behavioral responses on a trial-by-trial basis? Is this possible? Such inclusion is not necessary in the present manuscript but it is likely that other readers will comment on this and therefore it may be something that could be mentioned in the discussion.

Issue 2

In the context of load theory, the manuscript discusses that the stronger representation of the masker is due to reduced cognitive load, assuming that the brain has more capacity to process the masker. While this interpretation is consistent with Lavie's load theory, which has been predominantly investigated in the visual domain, there might be additional considerations specific to the auditory domain. Extending the load argument further, would this imply that the stronger the masker is represented, the better?

The problem with this conclusion is that, in attentional terms, one might expect that a weaker representation of the masker would be a better indicator of successful attention or suppression of the distractor. If you split the dataset into two groups based on the median strength of the masker representation under noise reduction conditions, would you expect better performance for stronger masker representations?

Perhaps clarification in the discussion would address this issue.

Issue 3

As you refer to the phonological character of the masker and target, it might be worthwhile to include and briefly discuss the recent paper by Carta et al., Cortical encoding of phonetic onsets of both attended and ignored speech in hearing impaired individuals (PLOS ONE, November 2024). This paper explores cortical encoding of phonetic onsets in both attended and ignored speech, which could provide additional context for your findings.
