# Supplementary figures and images for: Improving Tracking of Selective Attention in Hearing Aid Users: The Role of Noise Reduction and Nonlinearity Compensation
Source: eNeuro. 2025 Feb 14;12(2):ENEURO.0275-24.2025. doi: 10.1523/ENEURO.0275-24.2025 (PMC11839092; doi:10.1523/ENEURO.0275-24.2025)

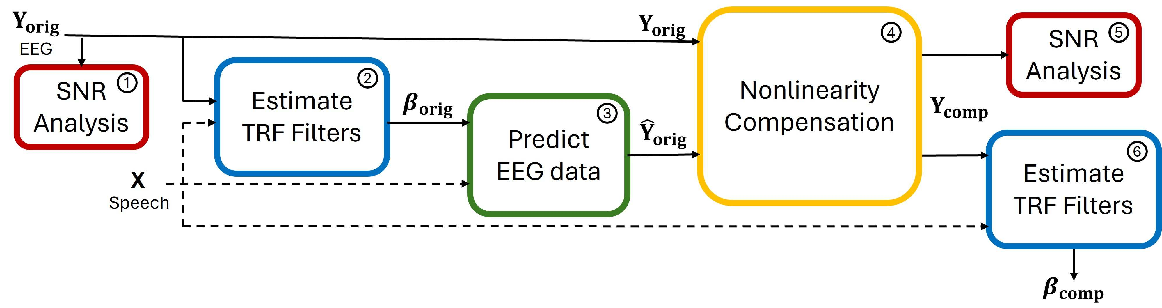

Supplement: Code — Download Code, ZIP file. [file eneuro-12-ENEURO.0275-24.2025-s006.zip › NonlinearCompensationEEG-main/Figures/figure1.png]

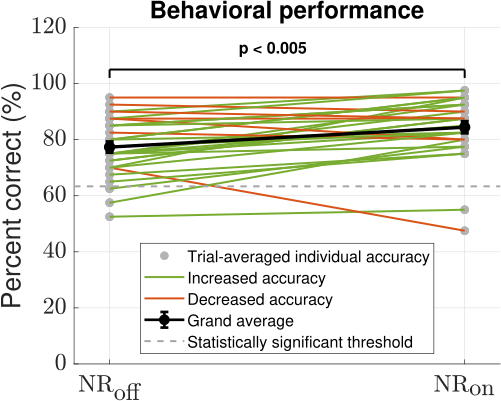

Supplement: Code — Download Code, ZIP file. [file eneuro-12-ENEURO.0275-24.2025-s006.zip › NonlinearCompensationEEG-main/Figures/figure10.png]

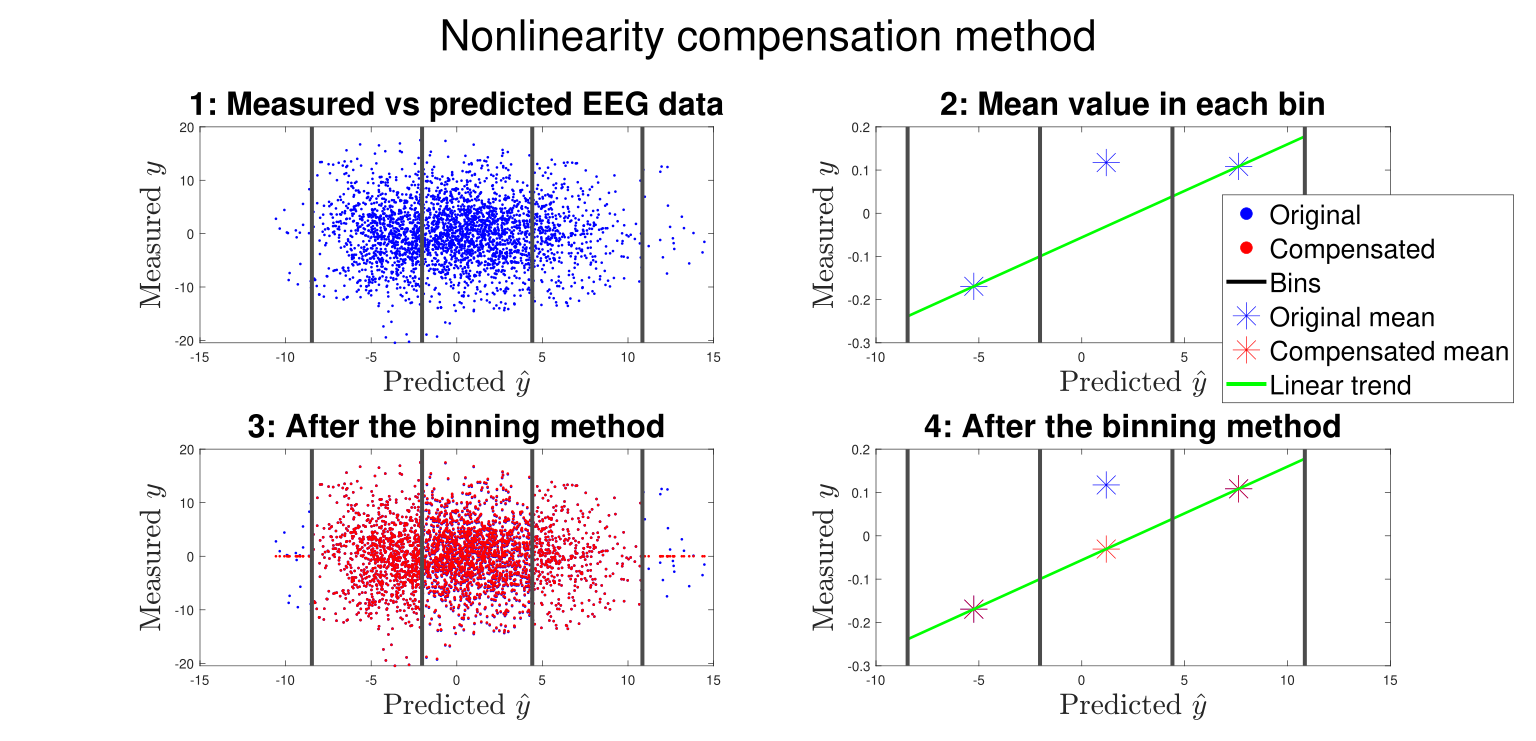

Supplement: Code — Download Code, ZIP file. [file eneuro-12-ENEURO.0275-24.2025-s006.zip › NonlinearCompensationEEG-main/Figures/figure2.png]

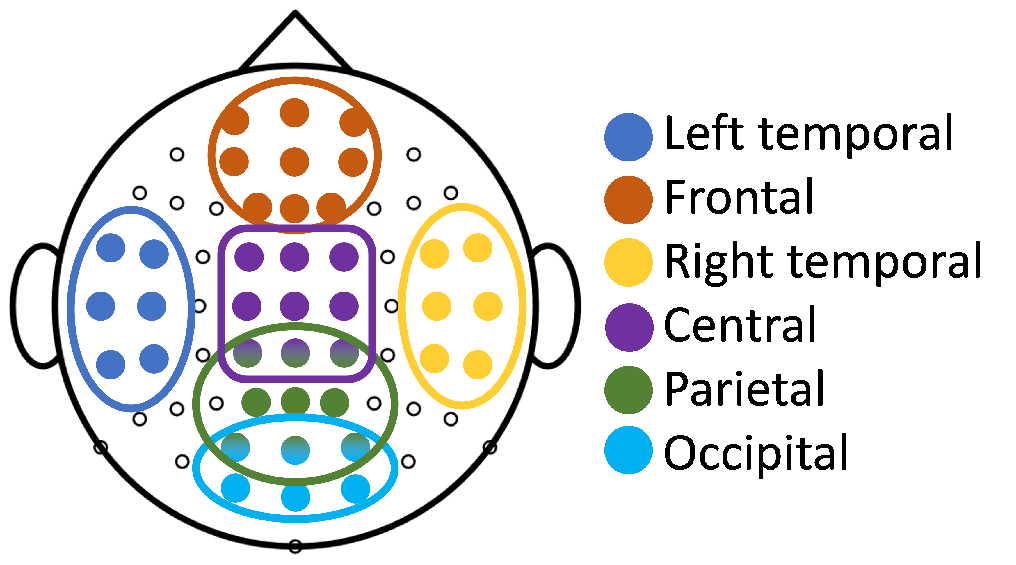

Supplement: Code — Download Code, ZIP file. [file eneuro-12-ENEURO.0275-24.2025-s006.zip › NonlinearCompensationEEG-main/Figures/figure3.png]

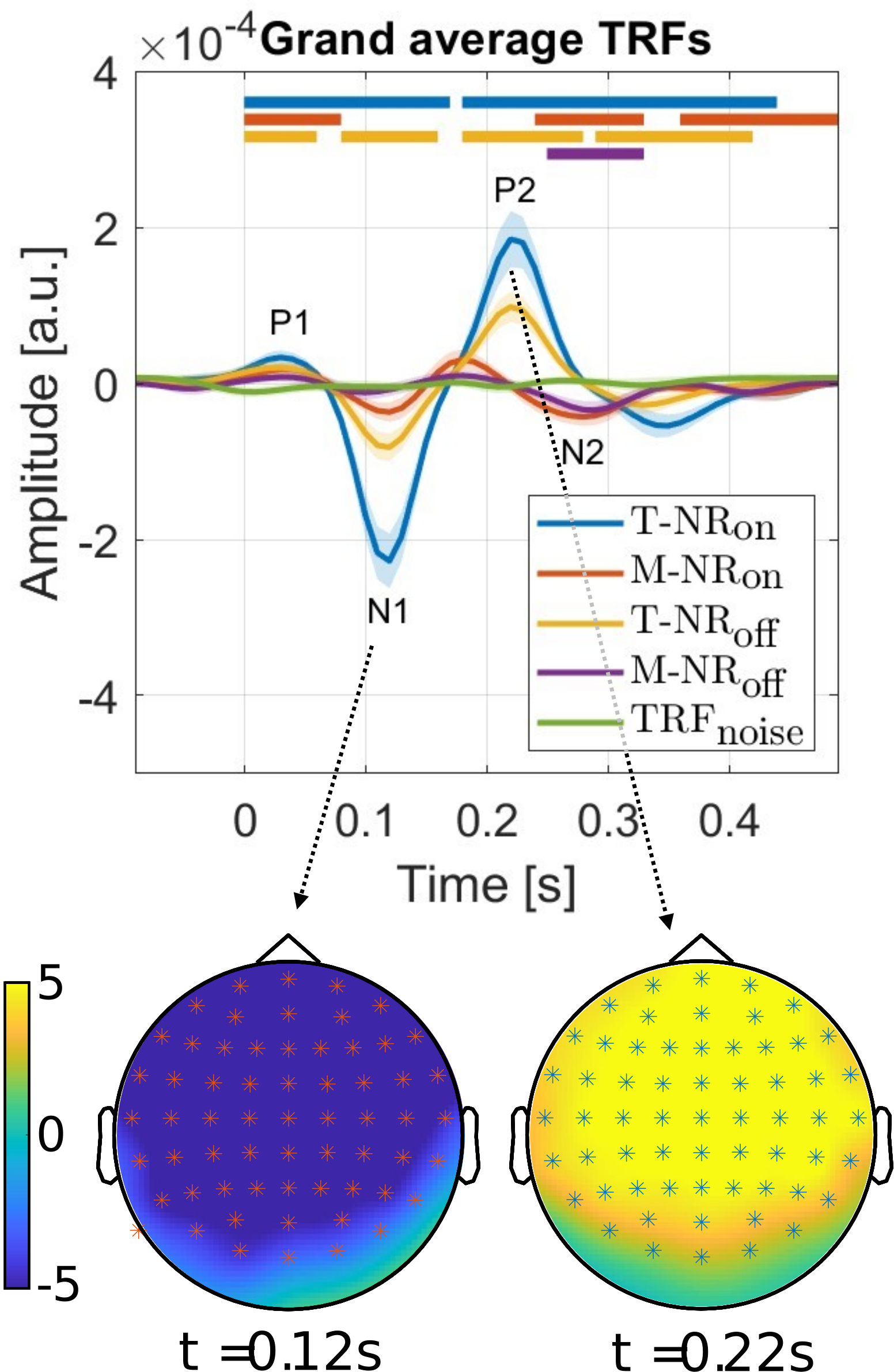

Supplement: Code — Download Code, ZIP file. [file eneuro-12-ENEURO.0275-24.2025-s006.zip › NonlinearCompensationEEG-main/Figures/figure4.png]

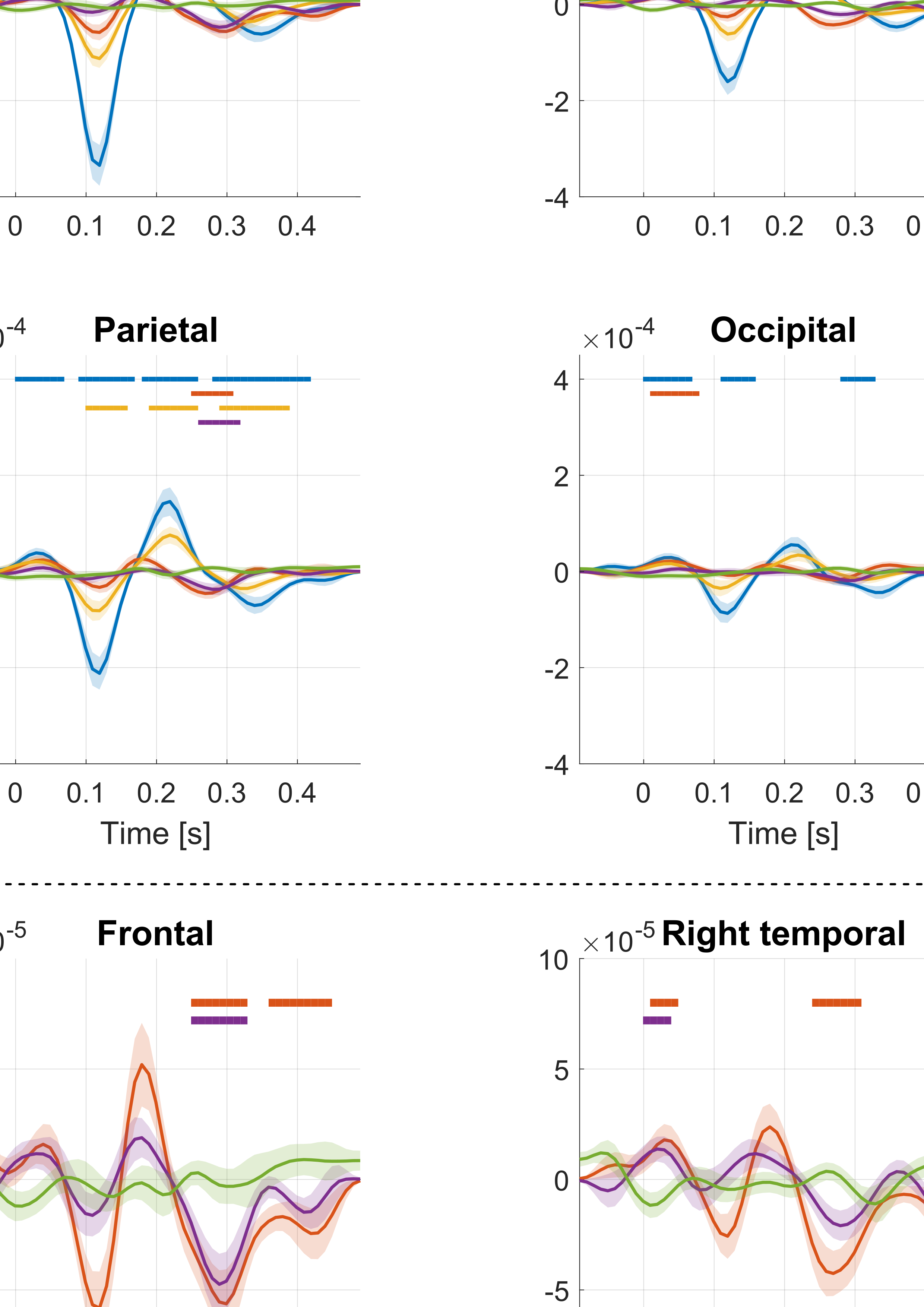

Supplement: Code — Download Code, ZIP file. [file eneuro-12-ENEURO.0275-24.2025-s006.zip › NonlinearCompensationEEG-main/Figures/figure5.png]

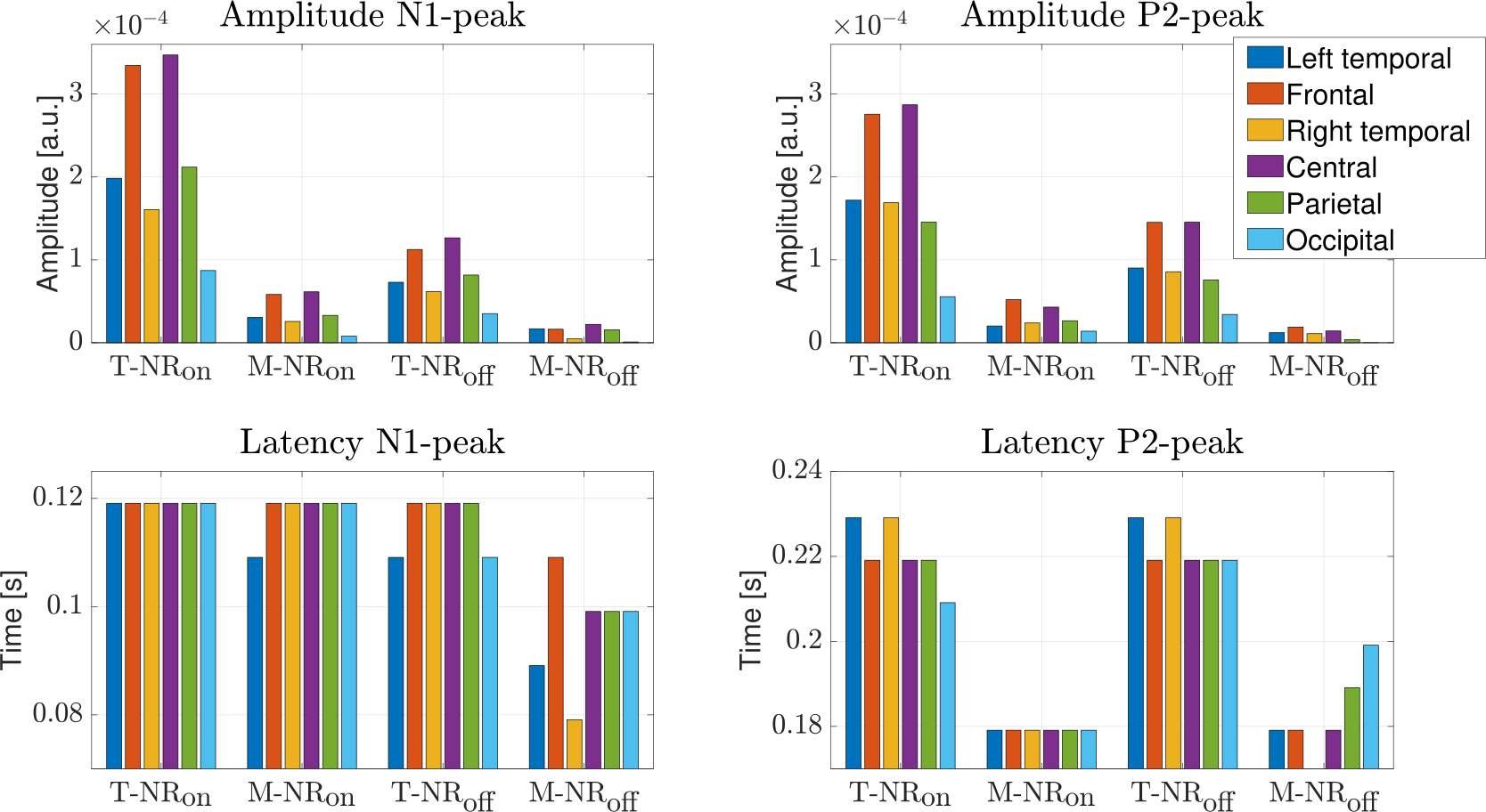

Supplement: Code — Download Code, ZIP file. [file eneuro-12-ENEURO.0275-24.2025-s006.zip › NonlinearCompensationEEG-main/Figures/figure6.png]

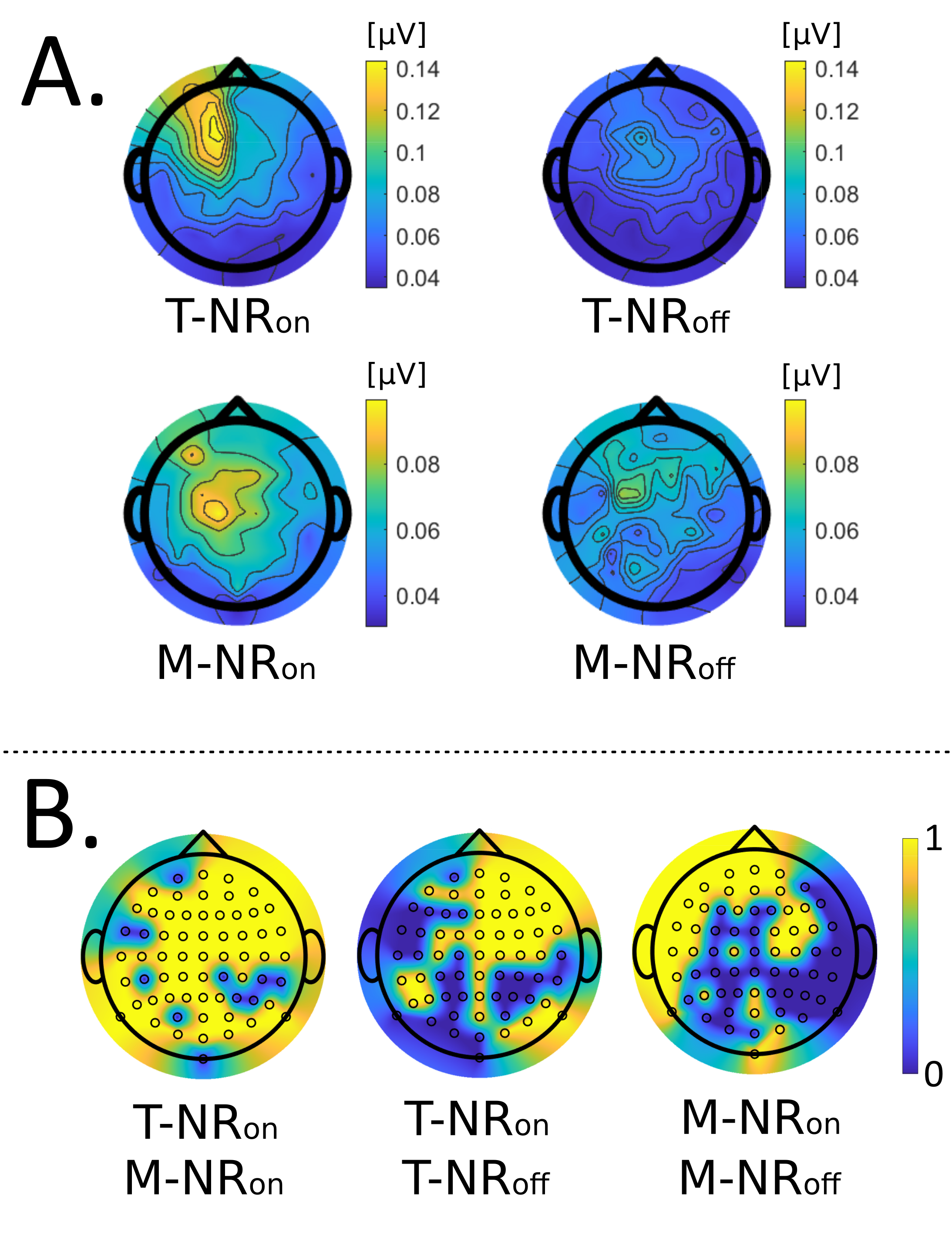

Supplement: Code — Download Code, ZIP file. [file eneuro-12-ENEURO.0275-24.2025-s006.zip › NonlinearCompensationEEG-main/Figures/figure7.png]

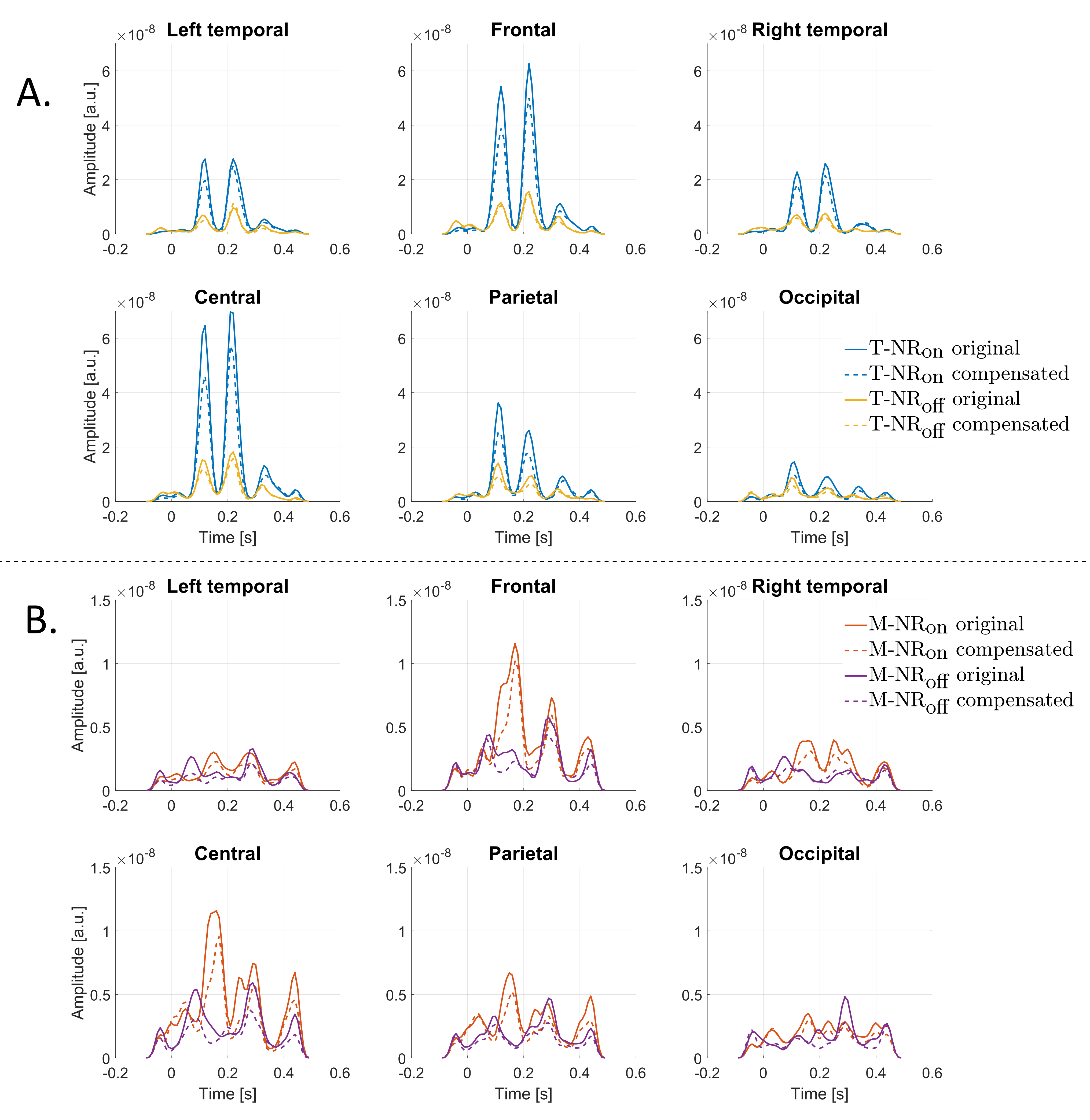

Supplement: Code — Download Code, ZIP file. [file eneuro-12-ENEURO.0275-24.2025-s006.zip › NonlinearCompensationEEG-main/Figures/figure8.png]

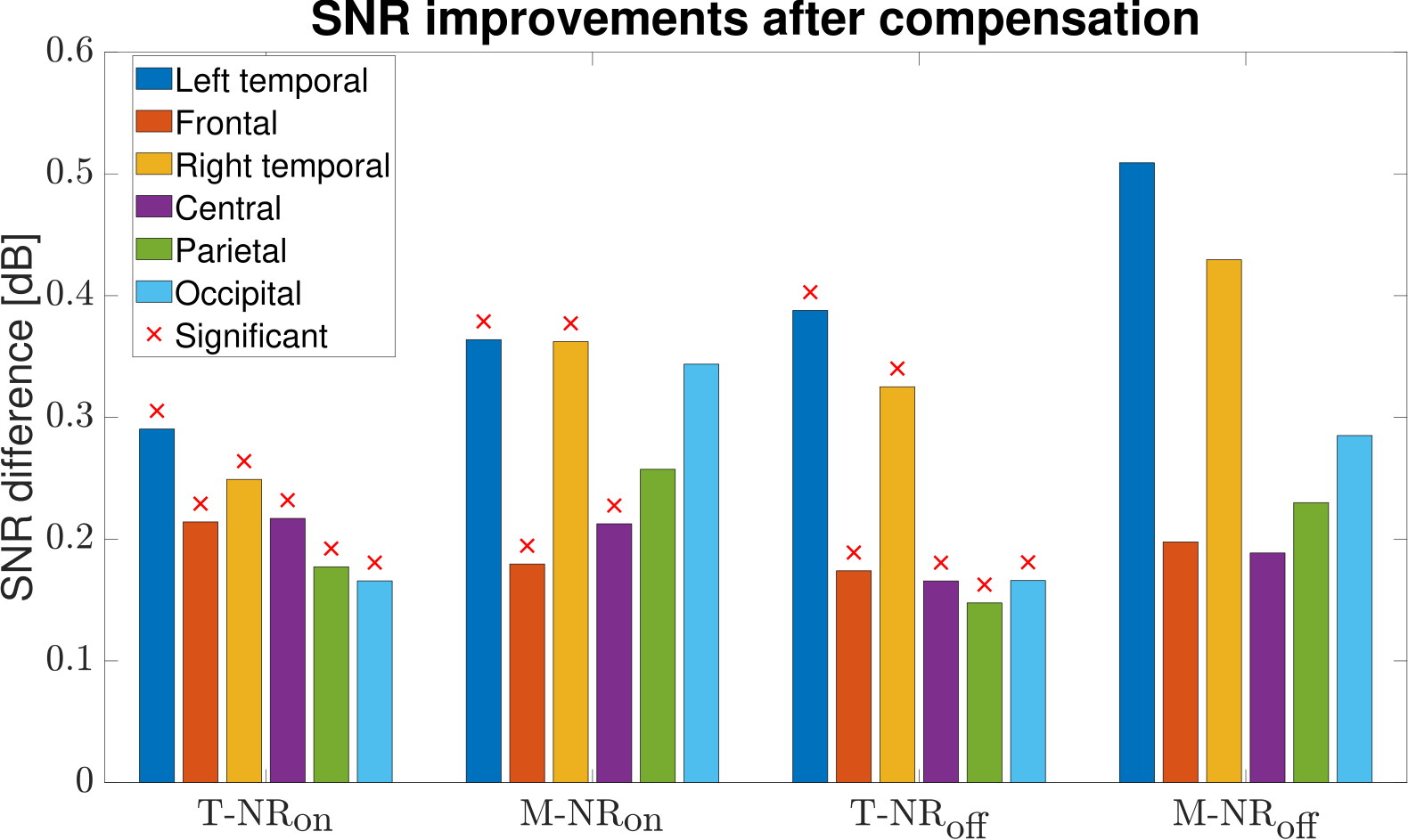

Supplement: Code — Download Code, ZIP file. [file eneuro-12-ENEURO.0275-24.2025-s006.zip › NonlinearCompensationEEG-main/Figures/figure9.png]

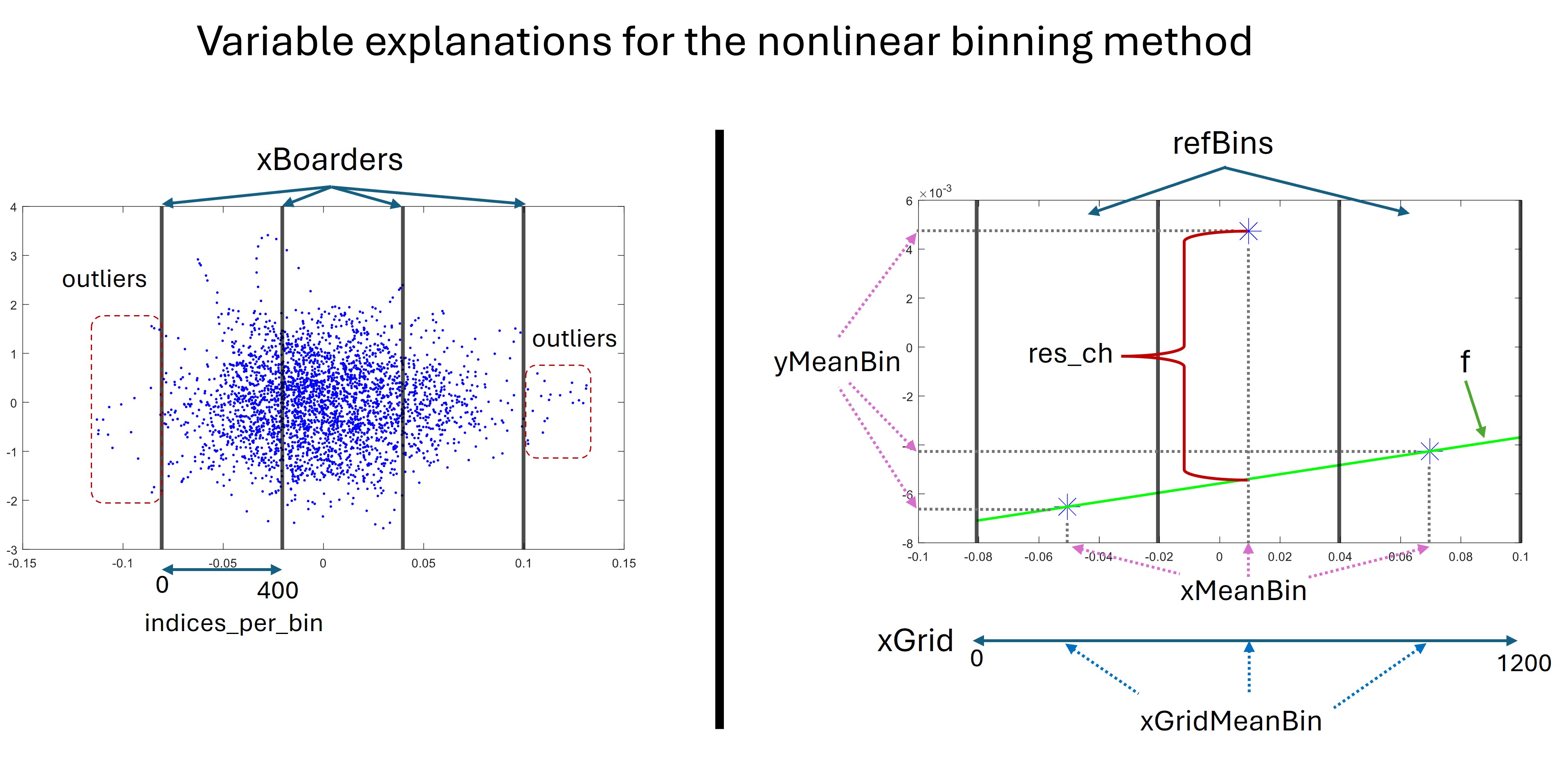

Supplement: Code — Download Code, ZIP file. [file eneuro-12-ENEURO.0275-24.2025-s006.zip › NonlinearCompensationEEG-main/Figures/variableExplanation_BinningMethod.jpg]
